# MXene‐Based Membranes for Separation Applications

**DOI:** 10.1002/smsc.202100013

**Published:** 2021-06-07

**Authors:** Lingzhi Huang, Li Ding, Haihui Wang

**Affiliations:** ^1^ Beijing Key Laboratory for Membrane Materials and Engineering Department of Chemical Engineering Tsinghua University Beijing 100084 China; ^2^ School of Chemistry and Chemical Engineering South China University of Technology Guangzhou 510640 China

**Keywords:** membranes, MXenes, separations, water treatments

## Abstract

MXenes are a new type of 2D material, featuring numerous favorable properties, such as excellent flexibility, hydrophilicity, abundant functional groups, and high conductivity. Currently, MXene is a hot topic in the field of membrane separation processes. This timely review presents the recent progress in MXene‐based membrane in terms of structural engineering and versatile applications. First, the preparation methods for MXene nanosheets and the fabrication technologies for MXene‐based membranes are categorized. Then, the separation‐based applications of MXene membranes, including gas separation, water treatment, organic solvent purification, nanofluidic ion transport, and osmotic energy conversion, are summarized. Finally, the bottlenecks that limit the application of MXene‐based membranes are outlined, and future research prospects are discussed.

## Graphical abstract

MXene, a new member of 2D materials, combining the merits of hydrophilicity, abundant surface groups, superb flexibility, and excellent conductivity, has been applied for high‐performance separation membranes. The recent progress and advancements in the applications of MXene‐based membrane are reviewed, as well as the discussion about the bottlenecks for further development.

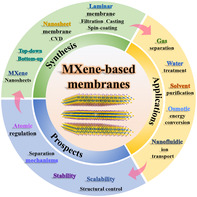

## 1 Introduction

With increasingly rapid industrial development over the last few decades, the separation process has been a procedure of prominent importance. Compared with traditional technologies, membrane technology combines the merits of lower energy consumption, higher efficiency, easier operation, and less secondary pollution,^[^ ^1^ ^]^ and has become one of the most promising candidates among numerous separation technologies. So far, the polymers are still the major materials for synthesizing separation membranes in wastewater treatment, large‐scale seawater desalination, food and pharmaceutical manufacturing, and hemodialysis‐based artificial kidneys.^[^ ^2^ ^]^ However, further development of polymer‐based membranes has been impeded by the trade‐off relationship between permeability and selectivity. This trade‐off concept was first proposed in the context that rubbery polymer membranes usually exhibit higher permeance whereas glassy polymers exhibit better selectivity.^[^ ^3^ ^]^

To obtain better separation performance, a series of advanced materials with superior properties have been investigated. Recently, 2D materials with atomic thickness have been extensively investigated as building blocks to prepare high‐performance membranes, which can be engineered on a nanometer or even sub‐nanometer scale. Henceforth, the transport resistance can be minimized to enhance the permeance and a more precise separation process can be ensured, indicating the great potential to break the trade‐off limitation. Typically, 2D material‐based membranes can be categorized into nanosheet membranes and lamellar membranes according to the formation of their channels.^[^ ^4^ ^]^ The former one denotes membrane comprises of single‐ or few‐layer nanosheets in which solutes are transported through the intrinsic or drilled pores in the nanosheets. Nanosheet membrane with atomic thickness is expected to reach ultimate permeance and selectivity. However, obtaining uniform and high‐density nanopores is still challenging. Numerous studies have shown that constructing lamellar membranes with stacked nanosheets is more practical for experimental operation. The transportation of molecules in laminated membranes is through the interlayer nanochannels and/or pores on nanosheets.^[^ ^5^ ^]^ Pioneered by graphene‐based membranes, these membranes have achieved unparalleled permeance and selectivity. For example, graphene oxide (GO) membranes with a thickness of 3–10 nm were fabricated by coating,^[^ ^6^ ^]^ showing extremely high CO_2_ permeability (8500 Barrer) and outstanding selectivity of CO_2_/N_2_ (20). Li et al.^[^ ^7^ ^]^ constructed ultrathin GO membrane with thickness from 1.8 to 20 nm through the vacuum‐assisted filtration (VAF), exhibiting unprecedented separation performance toward hydrogen with the selectivity of H_2_/CO_2_ and H_2_/N_2_ pair gases reached as high as 3400 and 900 and were quite stable under varying temperatures. The separation performance is higher than that of reported polymeric membranes, breaking through the upper bond of polymer membranes.^[^ ^8^ ^]^ Beyond GO, various 2D materials have been investigated for the preparation of separation membranes, such as molybdenum disulfide,^[^ ^9^ ^]^ metal‐organic frameworks (MOFs),^[^ ^10^ ^]^ covalent organic frameworks (COFs),^[^ ^11^ ^]^ and zeolites,^[^ ^12^ ^]^ bringing membranes to a span‐new 2D materials world. Recently, a new member of the 2D family, MXene, is capturing most of the attention of researchers due to its unique physiochemistry.

MXene was first synthesized by selectively extracting the A layers from MAX phases.^[^ ^13^ ^]^ MAX phases are typically composed of layered ternary carbides and nitrides with a formula of M_*n*+1_AX_*n*_, where M represents an early transition metal, A represents an IIIA or IVA element (Al, Ga, Si, and Ge), X represents carbon or nitrogen, and *n* varies from 1 to 3. The first and most‐studied MXene, namely, Ti_3_C_2_T_*x*_, was successfully exfoliated utilizing hydrofluoric acid (HF) as the etching agent. T represents the homogeneously terminated groups on the surface (such as oxygen, hydroxyl, and fluorine), and X represents the number of these functional groups.^[^ ^13^ ^]^ Due to preparation in acidic solution, MXene is endowed with a variety of exceptional properties, including high conductivity, good flexibility, abundant surface groups, hydrophilicity, and outstanding chemical stability. The natural hydrophilicity favors MXene to be applied in the membrane separation process whereas diverse functional groups enrich the structural tunability. Moreover, the stability of MXene‐based laminated membranes in the aqueous solution is prior to GO and other 2D materials, due to the weaker electrostatic repulsion and stronger van der Waals force between MXene adjacent nanosheets.^[^ ^14^ ^]^

**Figure 1:**
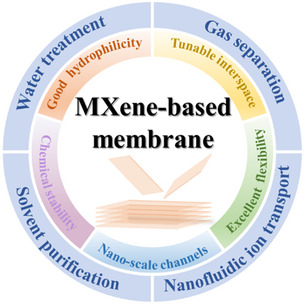
A summary of the advantages of MXene‐based membrane and its applications in the separation field.

After 10 years of development, MXene has been the new focus in energy storage,^[^ ^15^ ^]^ electronic,^[^ ^16^ ^]^ catalysis,^[^ ^17^ ^]^ and membrane applications.^[^ ^18^ ^]^ To meet the burgeoning need for MXene implementation in the aforementioned areas, researchers have extensively discussed the status, prospects, and future development of these materials.^[^ ^19^ ^]^ These discussions have made outstanding contributions to the development of MXenes, and the resulting publications have benefited many researchers and engineers by shedding light on future trends. However, among these review articles, only a few corresponds to membrane technology and gives a comprehensive introduction to MXene‐based membranes.^[19c,19e,19g]^ Meanwhile, reports about MXene‐based membranes have emerged rapidly and have shown that such membranes have achieved astonishing performance both in permeance and selectivity. Therefore, it is vitally important and necessary to review how MXene and MXene‐based membranes attain these incredible performances and gradually overcome the empirical trade‐off hurdle (**Figure** **1**).

In this review, we categorize the development skeleton of MXene‐based membranes, focusing on the most classical and latest progress, including the structural control and application in separation technology. First, the preparation methods for MXene are briefly discussed before we present the fabrication technologies for MXene‐based membranes. Second, the MXene‐based membranes that have been utilized for separation applications are summarized, covering gas separation, water treatment, organic solvent purification, nanofluidic ion transport, and osmotic energy conversion. In the end, we point out the limitations and prospects for the development of MXene in separation.

## 2 Synthesis of MXene

To date, over 30 kinds of MXenes have been successfully fabricated by diverse protocols, featuring different lateral sizes, defects, morphology, and properties. Nevertheless, although the processing parameters vary, these methods can be simply classified into two major categories: top–down and bottom–up. For the top–down strategy, the primary and leading method for producing MXene nanosheets is selectively etching the A layer (typically Al) from the parent MAX phase. The etching process renders the usage of highly corrosive etchants to prepare MXene nanosheets, which are usually accompanied by unavoidable defects and unevenly distributed surface‐terminated functionalities, whereas subsequent delamination and sonication would increase the number and size of these defects. In contrast, without etching, delaminating, or sonicating processes, MXene nanosheets fabricated by the bottom‐up method usually have fewer unintended defects and terminations. Moreover, the bottom‐up method has successfully prepared several kinds of new MXenes which are unable to be fabricated by the top–down method, for example, Mo_2_C.^[^ ^20^ ^]^ Specifically, the bottom‐up methods comprise chemical vapor deposition (CVD),^[^ ^20^ ^]^ template method,^[^ ^21^ ^]^ and plasma‐enhanced pulsed laser deposition (PEPLD).^[^ ^22^ ^]^

### 2.1 Top–Down

**Figure 2:**
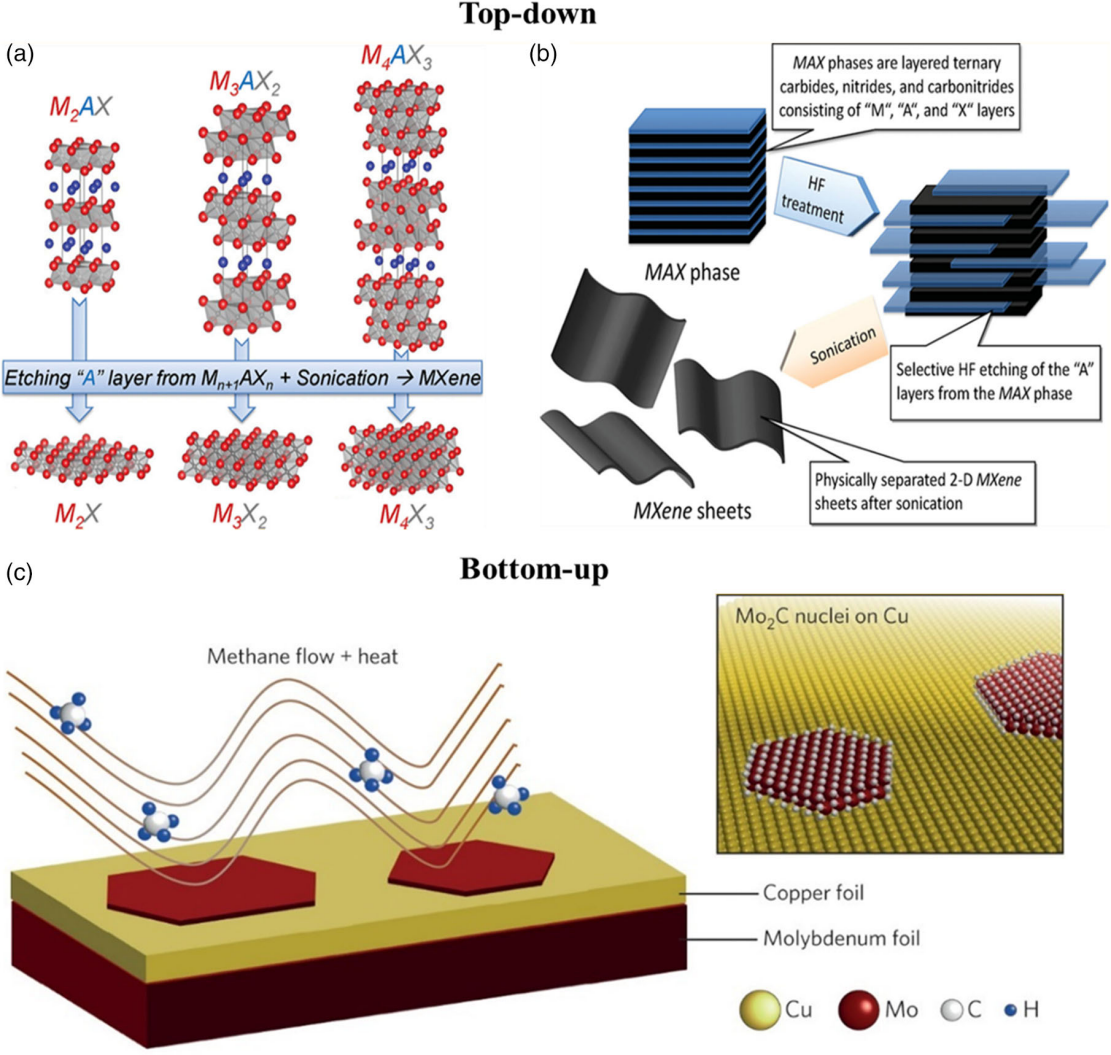
a) Illustration of different MAX phases and the corresponding MXenes. Reproduced with permission.^[^ ^70^ ^]^ Copyright 2013, Wiley‐VCH. b) The synthesis route of the top–down method. Reproduced with permission.^[23a]^ Copyright 2012, American Chemical Society. c) The bottom–up method to prepare Mo_2_C via CVD process. Reproduced with permission.^[^ ^48^ ^]^ Copyright 2015, Springer Nature.

As shown in **Figure** **2**a,b, the first step of top–down synthesis is etching[^19^, ^23^] in which the HF or in situ formed HF is used as the etchant. In 2011, the first member of the MXene family, Ti_3_C_2_T_*x*_, was synthesized after exposure to a concentrated solution of HF to chemically extract the Al layer from its precursor Ti_3_AlC_2_. Since the M—A bonding energy is lower than the M—X bonding energy, serial etching reactions can proceed smoothly step by step. Take Ti_3_AlC_2_ as an example^[^ ^13^ ^]^

(1) $${\text{Ti}}_{3}{\text{AlC}}_{2}+3\text{HF }\to {\text{Ti}}_{3}{\text{AlC}}_{2}+{\text{AlF}}_{3}+1.5H_{2}$$

(2) $${\text{Ti}}_{3}C_{2}+2H_{2}\text{O }\to {\text{Ti}}_{3}C_{3}{\left(\text{OH}\right)}_{2}+H_{2}$$

(3) $${\text{Ti}}_{3}C_{2}+2\text{HF }\to {\text{Ti}}_{3}C_{2}F_{2}+H_{2}$$

The as‐synthesized MXene is terminated with several typical functional groups (–O, –OH, and –F). These groups occupy the place of Al, which endows MXene with natural hydrophilicity and tunability compared to other 2D materials. However, it is commonly acknowledged that the HF solution is of extreme corrosivity and poses a great hazard to the operators and the environment. Therefore, the use of hydrochloric acid (HCl) and fluoride salt to form HF in situ, which was milder, began to attract researchers’ attention. In 2014, Chidiu et al.^[^ ^24^ ^]^ successfully synthesized titanium carbide “clay” combining the solution of lithium fluoride (LiF) and hydrochloric acid as etchant instead of concentrated HF solution. This method has been recognized as a milestone for expanding the MXene family due to its operability and safety. Moreover, due to the intercalation of these fluoride salts used in forming HF, MXene prepared by this method generally exhibits larger interlayer spacings, meanwhile speeding up the exfoliation process and reducing the etching time. In fact, in addition to HF or in situ formed HF, alkaline and molten salts have been gradually developed as etchants.^[^ ^25^ ^]^

The following step is delamination, to obtain single‐layer MXene nanosheets, in which intercalants such as dimethyl sulfoxide (DMSO)^[^ ^26^ ^]^ and tetrabutylammonium hydroxide (TBAOH)^[23b]^ are introduced to enlarge the interlayer spacings and weaken the interactions between the adjacent layers, which acts as a supplementary procedure to delamination.

### 2.2 Bottom–Up

It is worth noting that several kinds of MXene cannot be prepared via the top–down method up to now, for instance, MoC and MoN. Moreover, the inevitable terminated groups and defects of the nanosheets not only have a profound impact on the performance of the target application but also significantly impeded the exploration of the intrinsic properties of MXene. Comparably, the bottom–up method is more feasible to fabricate MXene nanosheets with fewer defects and better quality. For the very first time, Xu et al.^[^ ^20^ ^]^ fabricated defect‐free 2D ultrathin α‐Mo_2_C nanocrystals via the CVD method. This fascinating result was achieved by utilizing a bilayer substrate with a Cu foil sitting on top of a Mo foil, heated to a temperature above 1085 °C (the melting point of Cu), where methane serving as a carbon source (Figure 2c). Controlling the temperature at 1085 °C for 5 min, most Mo_2_C crystals feature a large lateral size of ≈10 μm and a relatively thin thickness of 3–20 nm. Initiated by Xu's report, the CVD process gradually broadened the MXene family. More than that, PEPLD, which is adapted from the CVD method, has also been demonstrated to be an effective approach to synthesize Mo_2_C films with tunable thickness.^[^ ^22^ ^]^

In the process of template method, 2D transition metal oxides are usually chosen to functioned as templates and the transformation of templates to MXenes is completed by carbonizing or ammoniating.^[^ ^21^ ^]^ Taking the synthesis of MoN for example, annealing the Mo precursor@NaCl under the protection of Ar atmosphere at first, followed by ammoniated by NH_3_, then after the removal of NaCl, the preparation of MoN nanosheets is completed.^[21a]^

### 2.3 Fabrication Methods for MXene‐Based Membranes

Due to their intriguing physicochemical properties, such as hydrophilicity, abundant surface groups, and flexibility, MXene nanosheets are advantageous to be constructed into membranes for separation process. So far, MXene‐based membranes reported for separation applications can be categorized into laminated MXene membranes and mixed matrix membranes.

Considering MXene‐based membranes are at the early stage of development, so majority of the membranes consist of pristine MXene nanosheets. To date, VAF is one of the most wildly accepted methods for preparing laminated MXene membranes. Homogeneously dispersed MXene nanosheets colloidal solution is strictly required if uniform membranes are anticipated. By varying the concentration or volume of the solution, membranes with different thicknesses can be obtained. Notably, the first separation membrane based on MXene was fabricated by Ren et al. in 2015,^[^ ^27^ ^]^ which was the pioneering achievement in the field of membranes. The free‐standing membrane was first fabricated by diluting delaminated Ti_3_C_2_T_*x*_ nanosheets evenly in deionized water and then filtered on commercial polyvinylidene fluoride (PVDF) support with a pore size of 450 nm. After completely drying, the MXene membrane with controllable thickness ranging from hundreds of nanometers to several micrometers was easily peeled off from PVDF substrate, exhibiting excellent flexibility and mechanical strength.

Apart from laminar stacked MXene membranes, MXene nanosheets also could be acted as filler in MMMs which could to decrease the mass transfer resistance, narrow the interlayer spacing, and engineer the transport performance.^[^ ^28^ ^]^ Introducing MXene nanosheets to relatively conventional matrix materials as additives or fillers greatly improves the feasibility to scale‐up and lower the cost. When constructing MMMs, the stability, uniformity, and compatibility of the fillers must be taken into consideration to maximize the advantages of MXene. In recent cases, blade casting and spin coating are more common strategies to fabricate MMMs.^[^ ^28^ ^]^ MXene nanosheets with a lateral size of 500–1000 nm and a thickness of 1–2 nm were incorporated into chitosan (CS) through spin coating for solvent dehydration.^[28c]^ Pandey et al.^[28b]^ used a traditional blade to cast the composite of MXene and cellulose acetate(CA) on a clean glass, to study the antifouling ability and selectivity of the MXene@CA membrane. Not surprisingly, the VAF method is also capable of preparing MMMs. For instance, Kang et al.^[^ ^29^ ^]^ fabricated a lamellar Ti_3_C_2_T_*x*_‐GO membrane with a thickness of 90 nm that was achieved by filtering the uniformly mixed Ti_3_C_2_T_*x*_ and GO solution on porous supports.

The fabrication method plays a dominant role when high‐performance separation membranes are desired. As high‐performance nanosheet membranes strictly require uniform and high‐density nanopores, we consider that drilling additional pores on originally defect‐free MXene nanosheets might be workable, nevertheless, limited by the scalability. For laminated membranes, a well‐stacked structure with superb stability is expected. The functional groups on the MXene surface play a significant role in regulating the stacking behavior, which determines electrostatic interaction of MXene nanosheets and the width of the nanochannels. So far, the MXene nanosheet synthesized by the top–down synthesis and self‐stacked via the VAF method is the most common assembly for the construction of the laminated membranes, given that chemical etching endows MXene with abundant functionalities and the thickness of the membrane as well as the defects (the thinner, the more defects are generated) could be tuned facilely via the VAF process. Simultaneously, MXene nanosheets with single layer and specific lateral size would be ideal for MMMs to seal the nonselective defects. As summarized in **Table** **1**, from the synthesis of MXene nanosheets to the fabrication of membrane, there are bunches of parameters available to be controlled, from the choice of the precursors, the type of etchants, the time of etching, the option of intercalants, and the use of sonication, to the fabrication of MXene membranes. Specifically, the structure of the precursor directly decides the quality of MXene sheets, whereas the etching process influences the separation of adjacent nanosheets and the surface functionalities. Although intercalant and sonication are auxiliary tools to facilitate exfoliation, the resultant MXene nanosheets might be monolayer with rather small lateral size and full of pores, leading to the loss of selectivity in separation. Henceforth, we can conclude that through precise control of the synthesis, we are able to regulate the separation performance of the MXene‐based membrane.

**Table 1:** The synthesis and performance of MXene‐based membranes in separation applications

MXene	Nanosheets	Membranes	Application	Ref.
Ti_3_C_2_T_*x*_	Method: LiF + HCl 35 °C 24 h	Method: VAF	Field: Gas separation	[18a]
	+ 10 min sonication	Thickness: 2 μm	Performance:	
	Lateral size: 1–2 μm	Channels: 0.35 nm	H_2_/CO_2_ selectivity 166.7	
	Number of layers: 1	Defects: None	H_2_ permeance: >2200 Barrer	
Ti_3_C_2_T_*x*_	Method: LiF + HCl 35 °C 24 h	Method: VAF	Field: Gas separation	[31]
	+ 60 min sonication	Thickness: 20 nm	Performance:	
	Lateral size: 1–2 μm	Channels: 0.52 nm	H_2_/CO_2_ selectivity: 27	
	Number of layers: 1 or 2	Defects: None	H_2_ permeance: 1584 GPU	
Ti_3_C_2_T_*x*_	Method: LiF + HCl 35 °C 24 h	Method: VAF	Field: Ion sieving	[27]
	+ 60 min sonication	Thickness: 1.5 μm	Performance:	
	Lateral size: hundreds of nanometers to several microns	Channels: 0.66 nm	Ion permeance: Na^+^ > Li^+^ > K^+^ > Ca^2+^ > Ni^2+^ > Mg^2+^ > Al^3+^ ≫ MB^+^	
	Number of layers: 1	Defects: None	Water permeance: 37.4 L/(Bar·h·m^2^)	
Ti_3_C_2_T_*x*_	Method: LiF RT 24 h + DMSO	Method: VAF	Field: Water treatment	[47]
	+ 20 min sonication	Thickness: 400 nm	Performance:	
	Lateral size: 100–400 nm	Channels: 2–5 nm	Effectively reject molecules larger than 2.5 nm	
	Number of layers: 1 or 2	Defects: Yes	Water permeance: 1000 L/(Bar·h·m^2^)	
Ti_3_C_2_T_*x*_	Method: HF 55°C 72 h	Method: VAF	Field: Solvent permeation	[18c]
	+DMSO + 10 min sonication	Thickness: 230 nm	Performance:	
	Lateral size: 3 μm	Channels: 2 nm	Reject molecules larger than 2 nm	
	Number of layers: 2	Defects: None	Organic solvents Permeance: 5000 L (Bar h m^2^)^−1^	
Ti_2_CT_*x*_	Method: LiF + HCl 35°C 24 h	Method: VAF	Field: Solvent dehydration	[64]
	Lateral size: 1–2 μm	Thickness: 100 nm	Performance:	
	Number of layers: 1 or 2	Channels: 0.44 nm	Water content: >99 wt%	
		Defects: None	Flux: 1069 ± 47 g (m^−2^ h^−1^)	
Ti_3_C_2_T_*x*_	Method: LiF + HCl 85°C 120 h	Method: VAF	Field: Solvent permeation	[71]
	+ 20 min sonication + freeze	Thickness: 2 μm	Performance:	
	Lateral size: 400–600 nm	Channels: 1.55 nm	Water permeance: 5460 L (Bar h m^2^)^−1^	
	Number of layers: 1 or 2	Defects: Yes	Acetone permeance: 3745 L (Bar h m^2^)^−1^	
Ti_3_C_2_T_*x*_	Method: LiF + HCl 35 °C 24 h	Method: VAF	Field: Antibiotics Separation	[54]
	+ 60 min sonication	Thickness: 500 nm	Performance:	
	Lateral size: 2–4 μm	Channels: 0.35 nm	Rejections over 90% for various antibiotics	
	Number of layers: 1	Defects: Yes	Permeance: 200–300 L (Bar h m^2^)^−1^	

## 3 Applications in Separation Processes

Possessing the merits of hydrophilicity, superb flexibility, and abundant surface groups, which highly align with the demands of separation membranes, MXenes have attracted enormous attention in fields involving separation processes and achieved outstanding performances. In this section, we will elaborate on recent progress relevant to the use of MXene‐based membranes in separation applications, namely, gas separation, water treatment, organic solvent purification, nanofluidic ion transport, and osmotic energy conversion.

### 3.1 Gas Separation

Ultrathin MXene nanosheets with single‐ or few‐layers can be easily obtained and act as building blocks to construct membranes with nanometer, even sub‐nanometer interlayer spacings, implying the enormous potential to precisely separate gas molecules based on the size exclusion mechanism. Till now, the application of the MXene nanosheets membranes with intrinsic or artificial pores in gas separation has been limited by the uniformity and scalability, so it is more feasible to utilize the laminated MXene membranes to separate gas. For the acquisition of high permeance and selectivity, the laminated membranes are rigorously required to have uniformly distributed sub‐nanometer channels without any defects.

**Figure 3:**
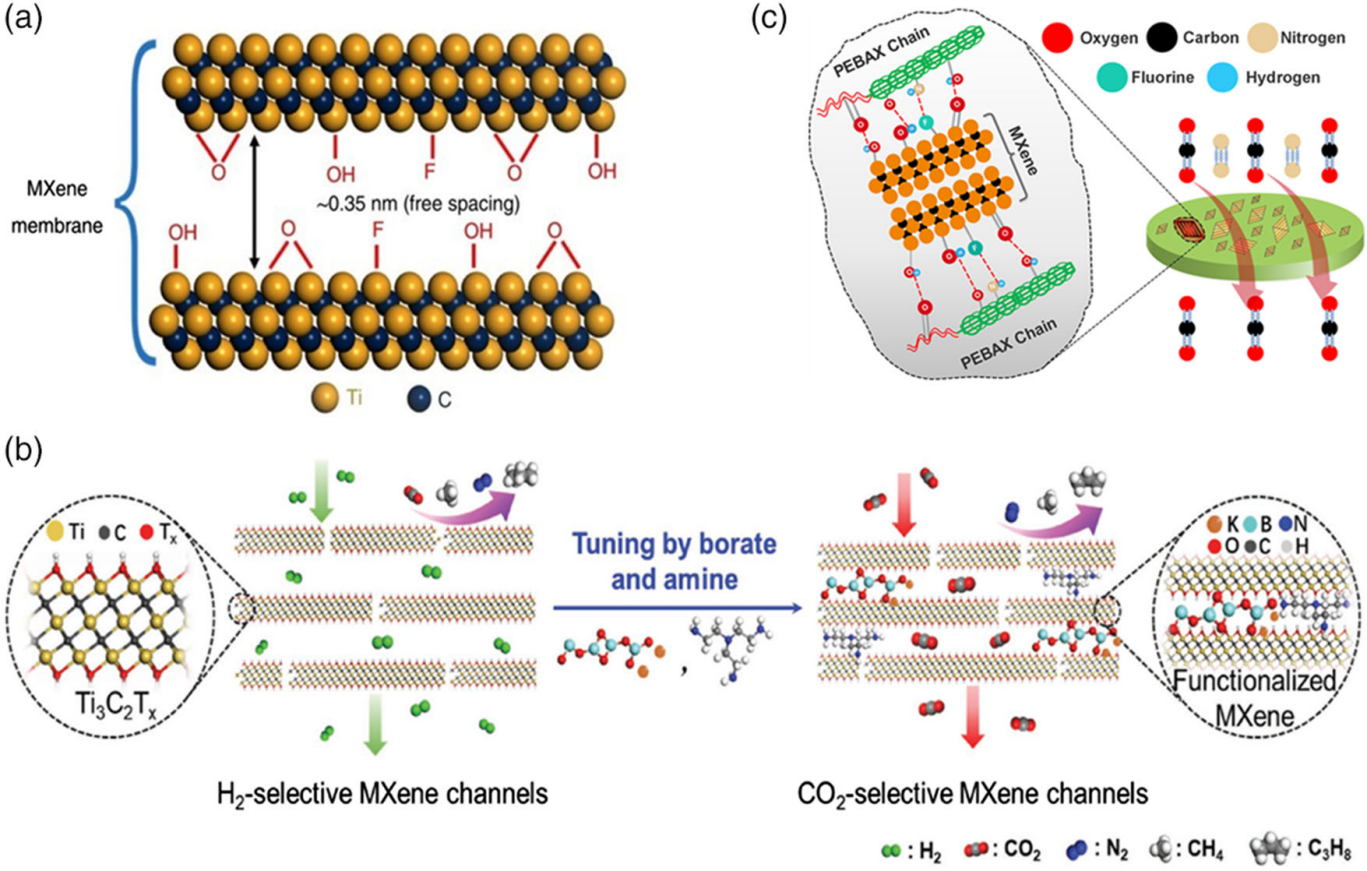
a) The channels with a critical width of 0.35 nm in the MXene laminated membrane for gas separation. Reproduced under the terms of the CC‐BY 4.0 license.^[18a]^ Copyright 2018, The Authors, published by Springer Nature. b) Schematic illustration of pristine MXene and functionalized MXene membrane specifically for H_2_ and CO_2_. Reproduced with permission.^[^ ^31^ ^]^ Copyright 2018, Wiley‐VCH. c) The structure of the MMMs and the interaction between MXene nanosheets and the matrix.^[^ ^34^ ^]^ Copyright 2019, American Chemical Society.

For the first time, our group^[18a]^ constructed a 2D laminar sieving membrane using exfoliated MXene nanosheets as building blocks to selectively separate gas molecules, obtaining ultrahigh permeance for H_2_ and excellent H_2_/CO_2_ selectivity. Prepared by the typical top–down method, MXene was exfoliated after etching by LiF and HCl. Then, MXene nanosheets, with a thickness of 1.5 nm and a lateral size of 1–2 μm, were chosen for the fabrication of MXene laminated membranes. Transmission electron microscopy (TEM) and X‐ray diffraction (XRD) analysis solidly confirmed the existence of highly regular sub‐nanometer channels and a well‐ordered laminar structure of the membrane with a 0.35 nm free spacing (**Figure** **3**a), which facilitated subsequent gas separation process. The as‐prepared MXene membrane showed good mechanical properties, with a tensile strength of over 50 MPa and Young's modulus of 3.8 GPa, and good reproducibility, laying the foundation for large‐scale membrane production. Governed by the gas kinetic diameter, the gas separation behaviors were highly consistent with the molecular dynamics (MD) simulations. For molecules with relatively small sizes, the as‐synthesized membrane demonstrated permeabilities of 2164 Barrer for He and 2402 Barrer for H_2_. The permeance drastically decreased for large molecules, for example, the permeability was only 10 Barrer for CO_2_. This intriguing phenomenon indicated that CO_2_ molecules have strong interaction with MXene because of their large quadrupole moment, thus inducing the sharply decreased permeability. Furthermore, the trapping effect of CO_2_ contributed to the unparalleled selectivity of H_2_/CO_2_ (about 166.6), paving the way for hydrogen purification and gas separation. Meanwhile, we systematically studied the gas transportation mechanism within MXene nanochannels through MD simulation, through which the diffusion features of He, H_2_, CO_2_, N_2_, and CH_4_ molecules were discussed.^[^ ^30^ ^]^ This work offered a valuable perspective for comprehensively understanding gas transportation behaviors in MXene‐based membranes and promoted the development of other 2D laminar membranes in gas separation.

Despite limited by the trapping effect, the permeation of CO_2_ had been improved by cross‐linking borate and polyethyleneimine (PEI) with MXene nanosheets to tune the interlayer spacing (Figure 3b).^[^ ^31^ ^]^ Adopting a subtly different method from previous work, MXene was filtered on a dopamine‐modified anodic aluminum oxide (AAO) porous substrate to enhance adhesion between MXene and the substrate. Therefore, a smooth and continuous membrane without any visible pinholes or defects was fabricated. With a critical thickness of 20 nm, the pristine MXene membrane showed outstanding H_2_ permeance of 1584 gas permeation units (GPU) and a high H_2_/CO_2_ selectivity of 27, transcending the 2008 Robeson upper bound^[^ ^8^ ^]^ for polymeric membranes. Considering the large interlayer spacing, which was not favorable for separating CO_2_ and CH_4_ (or N_2_), and the inevitable interactions between the CO_2_ and MXene surface groups, borate and PEI were integrated into the membrane to crosslink adjacent MXene nanosheets by bonding with oxygen functionalities. Thereout, not only the interlayer spacing was reduced but also the release of trapped CO_2_ was facilitated. The delicate functionalized MXene nanofilm demonstrated extremely high CO_2_ permeance (350 GPU), more than five times that of the pristine nanofilm, overwhelming upper bound for MOFs membrane.^[^ ^32^ ^]^

In practical situations, the thermal stability of membranes has a significant impact on operation and applications. MXene laminated membranes exhibited high‐temperature tolerance and stable gas separation performance at temperature up to 320 °C, reported by Fan et al.^[^ ^33^ ^]^ A moderate H_2_ permeance and selectivity of 41 for H_2_/N_2_ mixture was obtained at such high temperature. This was the first report that the 2D laminated membrane could work at such high temperature, which was distributed to the stable crystal structure of MXene and good thermal expansion matching with the substrate. Moreover, excellent sustainability was also found by continual testing over 200 h without the appearance of significant changes or apparent degradation. But when the temperature continued to increase, the selectivity started to decrease, signifying that more endeavors should be devoted to exploring the high‐temperature tolerance of MXene‐based membranes.^[^ ^33^ ^]^

It is notable that MMMs filled with the MXene nanosheets were also prepared for gas separation, making full use of the adsorption ability of MXene toward gases and additional channels provided by the introduction of the nanosheets.[^28^, ^34^] Liu and co‐workers^[28a]^ fabricated MMMs using Pebax as the matrix whereas MXene as the nanofillers. When the loading of MXene increased to 0.15 wt%, the permeance of CO_2_ was elevated by 81%, and the selectivity of CO_2_/N_2_ was elevated by 73%, achieving comparatively high CO_2_ permeance of 21.6 GPU and CO_2_/N_2_ selectivity of 72.5. Shamsabadi et al.^[^ ^34^ ^]^ embedded Ti_3_C_2_T_*x*_ MXene nanosheets in Pebax‐1657 with lower loading to synthesize thin‐film MMMs. Corroborated by characterization and MD simulation, hydrogen bonds formed between Pebax and MXene (Figure 3c). This bond formation attributed to the better performance with CO_2_ permeance of 1986.5 GPU and CO_2_/N_2_ selectivity ≈42.^[^ ^34^ ^]^ Furthermore, this improvement promised a more economical way to capture CO_2_ at the cost of $29/ton.^[^ ^35^ ^]^ More recently, the permeance of CO_2_ and the selectivity of CO_2_/N_2_ gas pair both have been greatly improved by Guan et al., given that the resulting permeability of CO_2_ was ≈86 Barrer and the CO_2_/N_2_ selectivity was over 104.^[^ ^36^ ^]^

### 3.2 Water Treatment

Terminated with versatile groups, MXene is negatively charged and hydrophilic, which is desirable to be applied in an aqueous environment for ion sieving and water purification. In addition, the surface functionalities favor MXene nanosheets to be chemically engineered to control the interlayer channels to separate various molecules of different sizes and to increase the permeability. According to the size‐exclusion mechanism, laminated membranes could sieve solutes larger than the channels whereas permeate the smaller ones. But if the solutes are charged, they can also be separated via the Donnan exclusion. Based on the electrostatic interaction, the ions with negative charges will be rejected by MXene whereas the passage of ions with positive charges will be facilitated. Thus, appropriately manipulate the surface chemistry of MXene can engineer the transport behavior and the separation performance. In this section, we will introduce the exceptional ion sieving capability of MXene‐based membranes at first and then the applications of the MXene‐based membranes in water treatment will be described from two angles: desalination and wastewater purification. At last, the emerging nanofluidic devices and the acquisition of osmotic energy based on the MXene membrane are given in brief.

#### 3.2.1 Background: Ion Sieving

**Figure 4:**
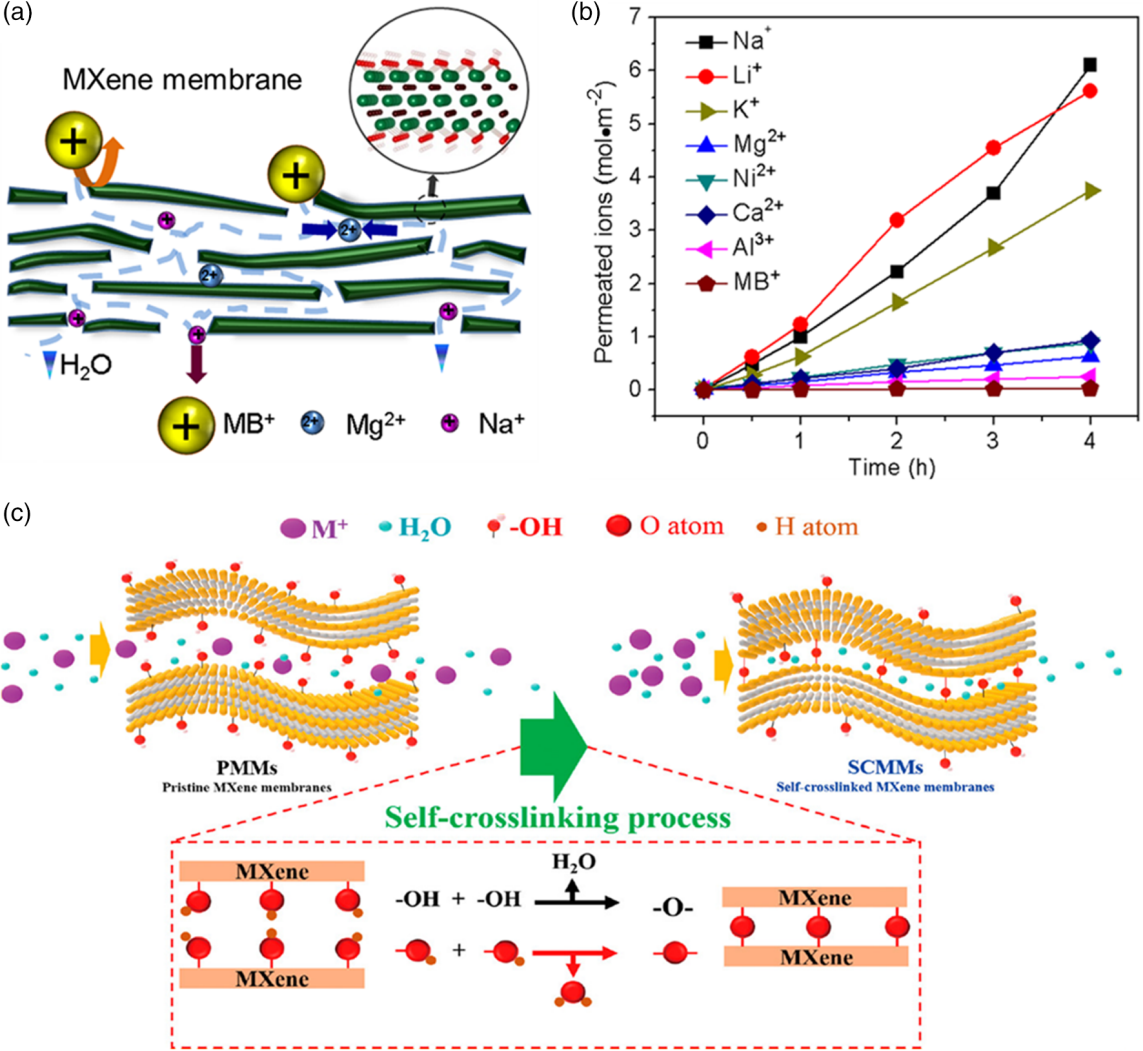
a) Schematic picture of MXene membrane sieving. b) Various cations permeation rates through the MXene membrane. a,b) Reproduced with permission.^[^ ^27^ ^]^ Copyright 2015, American Chemical Society. c) Illustration of the self‐cross‐linking process. Reproduced with permission.^[^ ^38^ ^]^ Copyright 2019, American Chemical Society.

The sieving ability of MXene was first explored in 2015,^[^ ^27^ ^]^ paving the road for separation applications in solutions (**Figure** **4**a). In this pioneering study, the MXene membrane was fabricated by the VAF method, with nanochannel diameters of ≈6.4 Å. Controlled at the same concentration, a series of monovalent and multivalent salt solutions were permeated through the MXene membrane, and the permeation rates were ranked as follows: Na^+^ > Li^+^ > K^+^ > Ca^2+^ > Ni^2+^ > Mg^2+^ > Al^3+^ (Figure 4b). MXene membrane showed great separation ability toward these salt ions, with the highest permeation of 1.53 mol h^−1^ m^−2^ for Na^+^, which was sevenfold of that of Ca^2+^ and 25‐fold of Al^3+^. Furthermore, MXene successfully overwhelmed GO in the selectivity of multivalent ions smaller than 4.5 Å. However, due to the strong interactions, water molecules and ions tend to intercalate between adjacent interlayers, known as swelling.^[^ ^27^ ^]^ At an early stage, researchers proposed that incorporating MXene with other existing technically mature materials to fabricate composite membranes could alleviate the swelling. Although ions were partially rejected by Donnan exclusion, the GO‐Ti_3_C_2_T_*x*_ composite membrane still maintained low rejection rates of salt ions (Na^+^ and Mg^2+^). These rates were no higher than 11%, considering that the enlarged space between two neighboring nanosheets failed to exclude small ions.^[^ ^29^ ^]^ Similar failure was demonstrated by the MXene/PES composite membrane.^[^ ^37^ ^]^

To this end, we proposed a novel concept—self‐cross‐linked MXene membranes, referring to the constrained interlayer spacing of MXene lamellas.^[^ ^38^ ^]^ Through a simple thermal treatment, a self‐cross‐linked MXene membrane was synthesized for metal ion rejection, by forming Ti—O—Ti bonds between adjacent interlayers to stabilize the interlayer space benefiting from considerable –OH groups terminated on MXene nanosheets. The dehydroxylation process (Figure 4c) was successfully conducted via the following reaction: –OH + –OH = –O– + H_2_O. As expected, the self‐cross‐linked membrane showed terrific antiswelling property and relative stability during 70 h operation, and desirable permeation rates of metal ions were obtained.^[^ ^37^ ^]^ In another related literature, a sodium alginate (SA) hydrogel homogeneously anchored on MXene nanosheet surfaces was introduced to form pillars in adjacent interlayers after the completion of the cross‐linking process by immersion in Ca^2+^, Ba^2+^, Mn^2+^, and Al^3+^ solutions.^[^ ^39^ ^]^ The pillared membrane exhibited outstanding rejection performance and ion sieving ability, with a nacre‐shaped architecture that was capable of restricting swelling in aqueous solutions and constraining interlayer space at 7.4 ± 0.2 Å. Specifically, based on different interactions of these multivalent cations with MXene, the Ca‐alginate pillared MXene membrane showed a prominent permeation cutoff, as well as ion selectivity, without any deterioration for 20 days, whereas the Mn‐alginate pillared membrane had high permeation with 100% rejection of Na_2_SO_4_.

Taking advantage of the excellent conductivity of MXene and the negatively charged surface, applying an external voltage has been proven to be an efficient strategy for improving the rejection rates toward ions and dyes^[^ ^40^ ^]^ of which we will give some examples in Section 3.4.

Herein, we can see that versatile strategies have provided new and efficient approaches for enhancing the ion selectivity and antiswelling properties of the membranes, assuring better utilization of MXenes in separation applications.

#### 3.2.2 Application: Water Desalination

In practical applications, apart from the high rejection rates of salts, desalination also requires long‐term stability. Recently, we introduced Al^3+^ ions to intercalate between MXene lamellae by concentration diffusion to form Al—O bonds,^[^ ^41^ ^]^ which efficaciously constrained the swelling of the MXene membrane in aqueous solution. Subsequently, the permeation tests for K^+^, Na^+^, Li^+^, Ca^2+^, and Mg^2+^ ions were conducted, and the Al^3+^ intercalated MXene membrane exhibited two orders of magnitude lower permeation rates compared with untreated MXene membrane, indicating the outstanding ion‐rejection performance of the treated MXene membrane. In addition, the Al^3+^ intercalated MXene membrane showed excellent stability during continuous operation for over 400 h, where mixed synthetic seawater (containing NaCl, Na_2_SO_4_, KCl, CaCl_2_, and MgCl_2_) was used as the feed solution to simulate the real‐life application. The water flux and salt rejections were conducted by forward osmosis under the transmembrane pressure of 49 bar. As the membrane thickness increased from 0.34 μm to 1.1 μm, the rejection of NaCl steadily remained above 90% with the sacrifice of water flux (decreased from 8.5 L m^−2^ h^−1^ to 2.8 L m^−2^ h^−1^).

In addition to the manipulation of intercalation, the desalination performance of MXene membranes can also be engineered by adjusting the interlayer spacing. This manipulation could be achieved by simply tuning the sintering temperatures without introducing external substances or incorporating other materials. Using tubular α‐Al_2_O_3_ as the support, MXene membranes were sintered in the range of 200 to 500 °C, and the permeance and rejection rates were evaluated under cross‐flow conditions.^[^ ^42^ ^]^ As the temperature increased, the permeance declined initially and later soared, whereas the rejection rates increased initially and later decreased, which could be attributed to the formation of oxygen bonds in the place of previous –OH groups when the temperature increased from 200 to 400 °C, leading to interlayer spacing shrinkage. When the temperature further increased to 500 °C, the transformation of Ti_3_C_2_T_*x*_ to TiO_2_ gave rise to a less organized lamellar structure and an altered filtration model, from lateral interlayer filtration to conventional one‐way flow. The optimized temperature of 400 °C exhibited the best separation performance toward salt solutions, but the rejection rates were not satisfactory.

**Figure 5:**
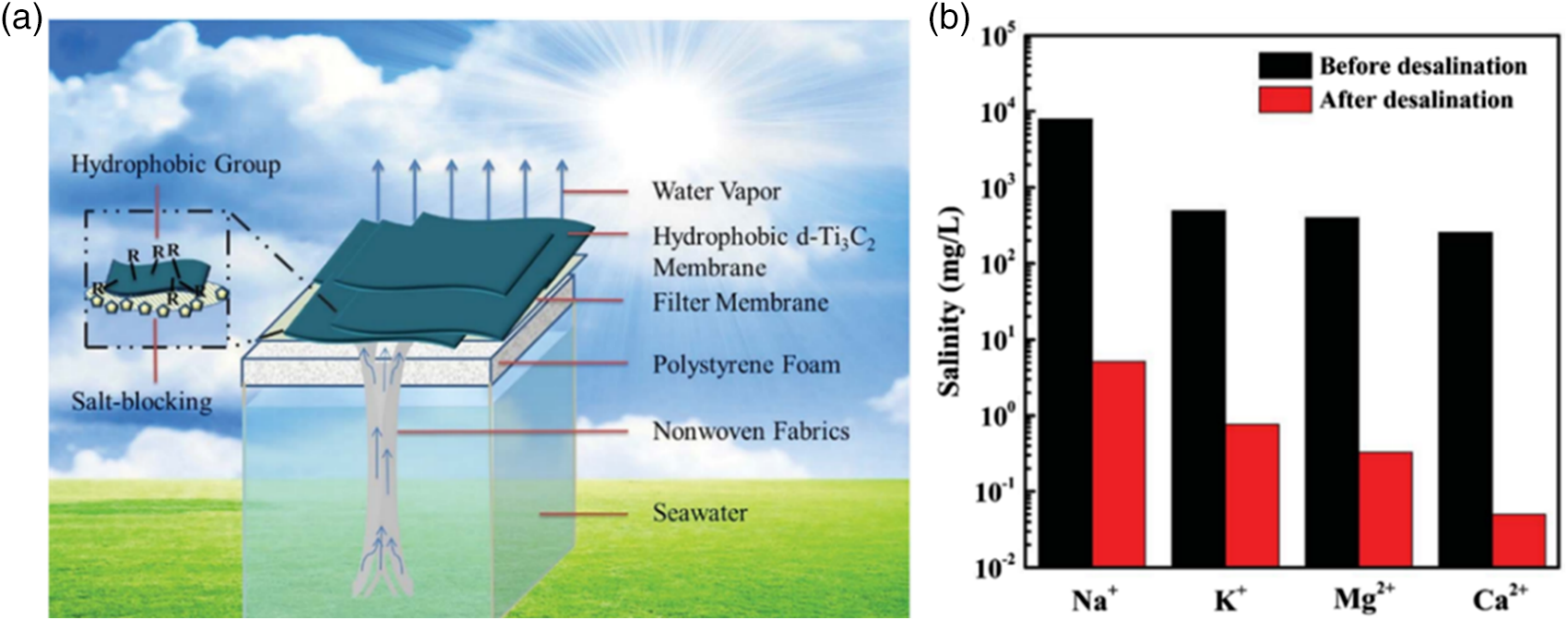
a) Schematic illustration of solar‐driven distillation device. b) MXene‐coated membrane's desalination performance of different salts. a,b) Reproduced with permission.^[^ ^43^ ^]^ Copyright 2018, The Royal Society of Chemistry.

In addition to the superb permeability and ion selectivity toward seawater, MXene membranes have the integrity of outstanding light‐to‐heat conversion ability, rendering them a hot spot in solar‐driven distillation. Traditionally, the membrane distillation process is a thermal‐driven process to pass the water vapor instead of liquid water, promoted by the vapor pressure difference across the membrane. In most solar‐driven distillation process, the MXene membrane is placed on the top of desalination device, serving as the heat absorber and vapor evaporator in which the water infiltrates the membrane through the capillary effect,^[^ ^43^ ^]^ shown in **Figure** **5**a. Through loading on porous and robust PVDF substrates, a polydimethylsiloxane (PDMS)‐modified self‐floating MXene membrane was used for the first time in a delicately designed photothermal evaporation system,^[^ ^44^ ^]^ achieving a 100% light‐to‐heat conversion efficiency and 74% light‐to‐water evaporation efficiency under 1 sun illumination. In addition, the workers utilized polystyrene (PS) foam as a heat barrier to optimize the surface structure; thus, the membrane demonstrated a higher light‐to‐water efficiency of up to 84%. Since real seawater consists of various salts that tend to crystallize on top of the membrane during evaporation, a hydrophobic MXene membrane modified by trimethoxy (1H, 1H, 2H, 2H‐perfluorodecyl) silane (PFDTMS) was proposed to efficiently block salts and prevent salt crystallization (Figure 5b), conferring the enhanced desalination performance with over 99.5% rejection rates for salt ions. Meanwhile, this hydrophobic membrane displayed superb evaporation performance as well as extraordinary stability, maintaining the evaporation rate of 1.31 kg m^−2^ h^−1^ without any decline for over 200 h.^[^ ^43^ ^]^ Beyond that, coupling the remarkable photothermal and antifouling properties of MXene, researchers have measured the feasibility of MXene‐coated PVDF membranes for employing direct contact membrane distillation.^[^ ^45^ ^]^ As expected, the input heating energy decreased by 12% per distillate volume and the membrane exhibited a notable fouling mitigation compared with the pure PVDF membrane. Considering the practical applications of this technology, Zha et al.^[^ ^46^ ^]^ confirmed that MXene nanosheets played an essential role in improving the membrane's antibacterial ability, advancing the development of solar‐driven membrane distillation.

#### 3.2.3 Application—Wastewater Purification

**Figure 6:**
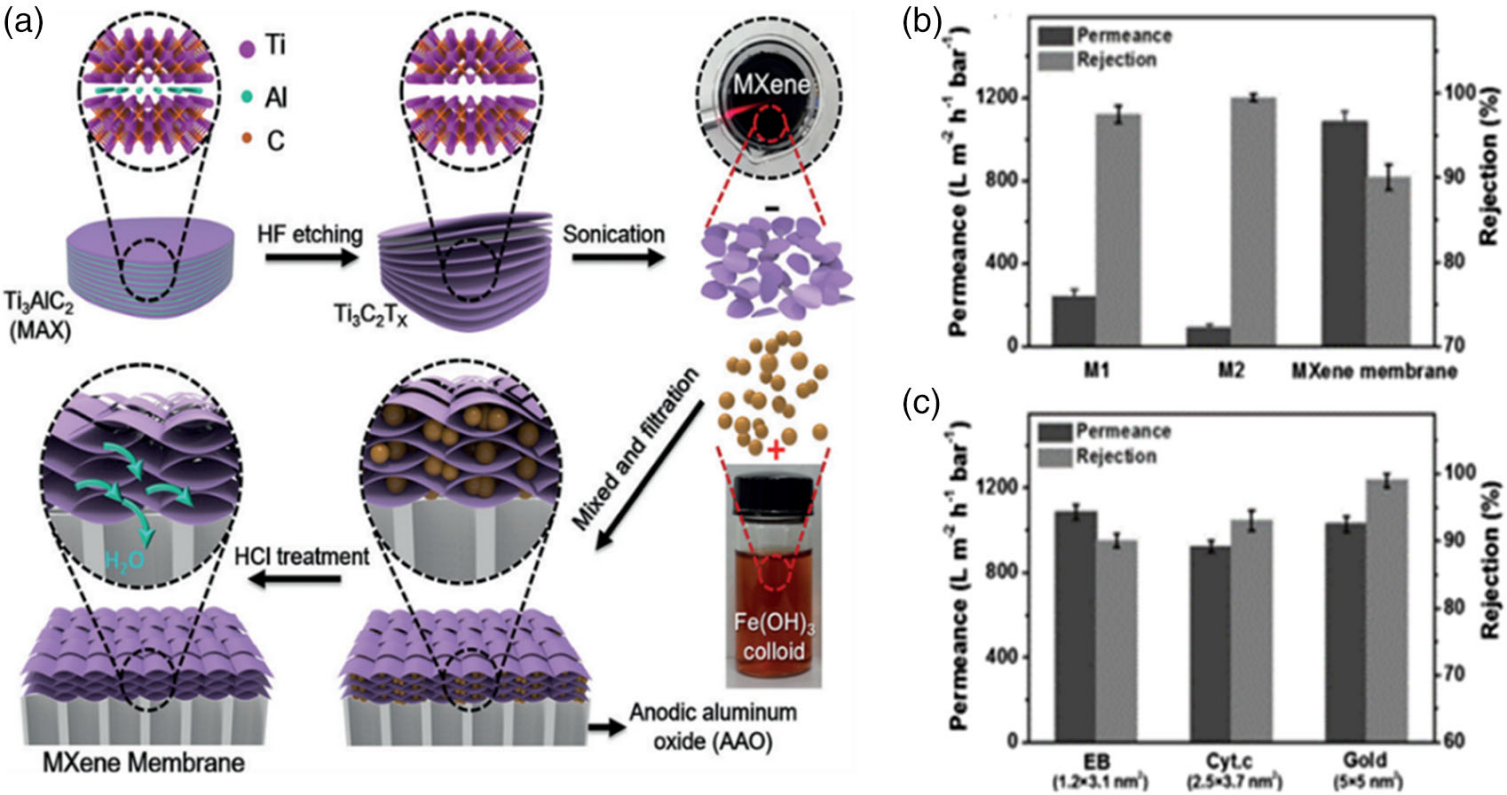
a) Fabrication of the laminar MXene membrane. b) EB separation performance comparison of the pure MXene membrane (M1), a composite membrane containing MXene and Fe(OH)_3_ (M2), and the as‐prepared MXene membrane for the separation of EB molecules at room temperature. c) This chart depicted that the MXene membrane demonstrated varying separation performance of EB, Cyt.c, gold. a–c) Reproduced with permission.^[^ ^47^ ^]^ Copyright 2017, Wiley‐VCH.

Our group first reported the utilization of MXene membrane in the water purification process.^[^ ^47^ ^]^ We innovatively fabricated porous and 2D lamellar Ti_3_C_2_T_*x*_ membrane via VAF assisted with the aid of Fe(OH)_3_ colloidal particles to expand nanochannels (**Figure** **6**a). On account that Fe(OH)_3_ nanoparticles and MXene nanosheets have opposite charges, Fe(OH)_3_ was bound to Ti_3_C_2_T_*x*_'s surface by electrostatic force, thus creating additional fluidic nanochannels that were advantageous for higher permeance, which had been verified by scanning electron microscopy (SEM) and energy‐dispersive X‐ray spectroscopy (EDX). Due to the abundant nanochannels and slightly enlarged interspace, the laminar MXene membrane achieved a water flux as high as 1084 L m^−2 ^h^−1 ^bar^−1^ (Figure 6b), meanwhile, maintained its structure intact and rather high permeance after immersed in water for over 1 month. Furthermore, the pore size of the Ti_3_C_2_T_*x*_ membrane was around 2–5 nm, in other words, the membrane failed to exclude molecules smaller than 2 nm. Therefore, as shown in Figure 8c, the membrane achieved almost complete rejection of gold nanoparticles (5 nm), whereas exhibiting 97% rejection for cytochrome c (Cyt.c 1.7 × 1.7 nm^2^), 90% rejection for Evans blue (EB, 1.2 × 3.1 nm^2^), and 85% rejection for rhodamine B (RB, 1.8 × 1.4 nm^2^).^[48a]^

For the sake of higher dye rejection rates, Ti_3_C_2_T_*x*_‐GO composite was selected to ultrathin and regular stacked membranes free of interedge nanopores or voids, inhibiting the nonselective permeance of the targeted small dyes.^[^ ^29^ ^]^ Commonly prepared through the VAF method, a 90 nm thick composite membrane with an interlayer spacing of ≈5 Å demonstrated efficient rejection performance of molecules with hydrated radii above 5 Å. As expected, almost complete rejection rates were obtained for methylene blue (MB) and brilliant blue (BB) of which the hydrated radii are above 5 Å. But for methyl red (MR) with hydrated radii of 4.87 Å, the rejection rate sharply decreased to 61%. However, the rejection of rose bengal (RosB, hydrated radii of 5.88 Å) was not ideal as MB and BB because of the weaker electrostatic interaction between MXene and the same negative‐charged RosB. Excitingly, higher rejection rates of organic dyes, as well as several typical natural organic matters, were attained in a similar work, demonstrating the positive synergistic effect of combining GO and MXene.^[^ ^49^ ^]^ Prepared by filtration on a mixed cellulose ester (MCE), GO–MXene composite membrane with a mass ratio of 1/4 was found to reach nearly 99.5% rejection for neutral red (NR), MB, crystal violet (CV), BB, and almost complete removal of humic acid (HA) and bovine serum albumin (BSA). Moreover, the water permeance of this composite membrane had profoundly increased, which was 11‐fold of that of the pure GO membrane, due to the increased nanocapillary channels after the introduction of MXene into the GO laminated membrane. Notably, as the electrostatic repulsion between contiguous layers reduced and interaction enhanced, the composite membrane exhibited superior stability over 1 month without any distinct degradation in permeance and rejection.^[^ ^49^ ^]^

Despite the aforementioned reports demonstrated excellent rejection toward organic dyes, we cannot neglect the plummeting water flux, which indicated a serious compromise in the balance between permeability and selectivity. Beyond mixing GO with MXene, MXene was introduced to incorporate with TiO_2_ as an additive to seal potential defects and improve selectivity.^[^ ^50^ ^]^ Xu et al. assembled MXene dispersion and TiO_2_ hydrosol with different MXene loading from 0–1 wt%, followed by coating on a 4 channel α‐Al_2_O_3_ hollow fiber support and disc‐like support, respectively.^[50a]^ After aging for 12 h at 60 °C and calcined at 400 °C in air, the as‐prepared membranes exhibited lower transport resistance and better filtration performance, due to MXene served as floor tiles to block potential defects and consequently prevented TiO_2_ sol infiltration. Comparably, hollow fibers achieved higher flux as well as better rejection of dextran than disc‐like membranes; this discrepancy may result from extra defects and pinholes when the thinner layer formed on the disc macroporous support. Adopting the same scheme, Sun et al.^[50b]^ increased the loading of MXene onto TiO_2_‐MXene composite membranes (0–5 wt%) and systematically investigated the influence of processing parameters such as coating time, calcination time, and temperature. The authors attributed the elevated flux to the introduced MXene nanosheets enriched the pathways, from the previous one‐way flow pathway to the longitudinal–lateral nanochannels.

Since it is unavoidable that the membrane will suffer from fouling (bacteria and oil) issues, and it is not realistic or economical to replace the membranes frequently, it is necessary to develop antifouling membranes. The antibacterial activity of MXene was first investigated by Rasool et al.^[^ ^51^ ^]^ who demonstrated that direct contact with MXene would damage the cells of *Escherichia coli* and *Bacillus subtilis*, consequently, over 98% of the bacteria cell viability loss was observed within 4 h of exposure to the Ti_3_C_2_T_*x*_ nanosheets. The antimicrobial mechanism could be explained as follows: MXene nanosheets with sharp edges can absorb on the surface of bacteria and disrupt the cell membranes; moreover, the natural hydrophilicity of MXene favors the contact with bacteria in solution, facilitating the cell damage. Meanwhile, due to the abundant functionalities on the surface, MXene could form hydrogen bonds with the cell membrane to impede the intake of nutrition, henceforth, inhibit the growth. Based on this, a series of antibacterial tests were conducted to verify that the MXene‐based membrane was capable to inhibit bacteria growth. Interestingly, a fresh membrane exhibited a 73% antibacterial rate against *B*. *subtilis* and 67% against *E*. *coli*. Moreover, after oxidation in air, the aged membrane showed a much higher growth inhibition (99%) of these bacteria.^[^ ^52^ ^]^

**Figure 7:**
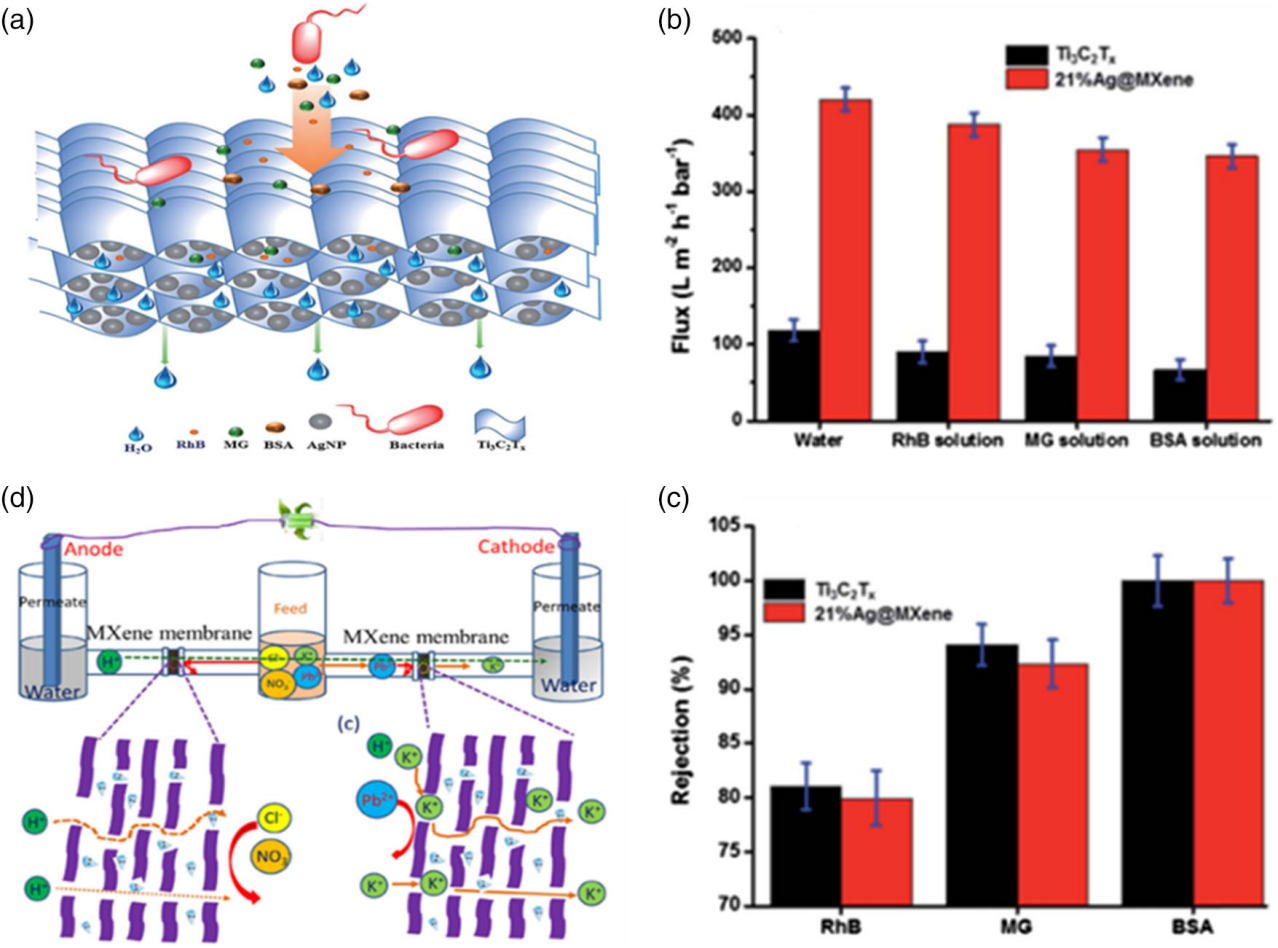
a) Antibacterial illustration of the Ag@MXene membrane. b) The total flux of Ti_3_C_2_T_*x*_ and 21% Ag@MXene membranes. c) Comparisons of rejection performance. a–c) Reproduced with permission.^[18b]^ Copyright 2018, The Royal Society of Chemistry. d) Schematic diagram of the Pb^2+^ sieving mechanism enhanced with an external voltage. Adapted with permission.^[^ ^57^ ^]^ Copyright 2020, Elsevier B.V.

To this end, Pandey et al.^[18b]^ introduced different loadings of Ag nanoparticles (AgNPs) to MXene (**Figure** **7**a) by the self‐reduction of silver nitrate on Ti_3_C_2_T_*x*_ nanosheets to achieve an ultrahigh growth inhibition of *E. coli* and simultaneously improved the water flux. Accordingly, they demonstrated that 21%Ag@MXene performed the best in terms of water flux (420 L m^−2^ h^−1^ bar^−1^) and bacterial inhibition rate (over 99% toward *E. coli*). The remarkably elevated permeance can be attributed to the additional nanopores that formed in the membrane due to the attached silver nanoparticles, whereas the enhanced inhibition was attributed to the synergistic antibacterial effects of AgNPs and MXene. Moreover, excellent organic molecule rejections were simultaneously reached without excessively sacrificing permeability. For example, almost perfect rejection of BSA was retained with similarly high permeance, but for relatively smaller ones (malachite green (MG) and RhB), a profound decrease in rejection rates was observed, as shown in Figure 7b,c. Recently, the same group further improved the rejection of MG (98%) and RhB (92%) by fabricating MXene/CA MMMs (designated MXene@CA),^[^ ^53^ ^]^ which could be ascribed to the formation of dense layers and chemical cross‐linking between MXene and CA.

To separate high‐added‐value drug in solutions, we utilized MXene nanosheets with high aspect ratio (thickness around 1.5 nm and lateral size of 2 μm).^[^ ^54^ ^]^ For the water‐soluble antibiotics tetracycline, erythromycin, azithromycin, and bacitracin, the MXene membrane showed promising rejections and maintained comparatively good permeance, especially for bacitracin, we observed a nearly complete rejection.

Oil fouling is also bothersome when superb stability and sustainability are desired. The ultrathin MXene membrane with a thickness of 30 nm showed excellent oil/water separation up to 99.94% as the oil concentration dropped to 10 ppm in the emulsion, whereas no apparent decay was observed after 50 cycles of testing for 25 h. Moreover, MXene membranes even exhibited a higher oil separation efficiency (>99.98%) for salt–oil water emulsions and retained outstanding recyclability, presumably due to the intercalation of salt ions narrowing down the interlayer spacing via electrostatic adsorption.^[^ ^55^ ^]^ Integrating printing technology into the preparation process of the MXene membrane was quite fascinating, where MXene ink was printed onto a low‐cost commercial paper. This facilely constructed membrane showed an outstanding removal rate for oil/water emulsions with oil concentrations less than 12 ppm but poor stability during the recycling experiment for only 8 cycles,^[^ ^56^ ^]^ far from the expected value.

A recent study reported a desirable rejection rate of Pb^2+^, up to 99%, as well as superior antiswelling.^[^ ^57^ ^]^Thermal cross‐linking was applied to effectively inhibit the swelling activity by drying the membrane at 70 °C to promote the cross‐linking process and engineer the interlayer spacing to 6.7–6.92 Å; moreover, an external voltage was introduced to facilitate the separation of noxious Pb^2+^ (8.01 Å) from Pb^2+^/K^+^ (6.62 Å) pairs. With the help of chemically cross‐linked sheets and external voltage, the separating factor reached 78 under an applied voltage of 16.5 V (Figure 7d).

### 3.3 Organic Solvent Purification

Pioneered by Wu et al.,^[^ ^58^ ^]^ MXene applications have been broadened in organic solvent nanofiltration through the incorporation with two conventional polymer matrices, hydrophilic PEI and hydrophobic PDMS. In contrast to pristine PEI or PDMS membranes, these polymer‐based membranes demonstrated a more robust structure and enhanced solvent resistance, presumably due to the large MXene nanosheets aligning with the matrix horizontally, thus inhibiting the motion of the polymer chain. Both PEI‐based and PDMS‐based membranes obtained greatly elevated isopropanol flux, exhibiting a 30% improvement for the PEI membrane and a 162% improvement for the PDMS membrane with only a 2 wt% embedment of MXene. Such satisfactory outcomes were attributed to the hydroxyl‐rich surface of MXene, on which hydroxyl groups functioned as adsorption sites by forming hydrogen bonds to transport isopropanol through the construction of laminar and longitudinal pathways. However, MXene nanosheets failed to interact with other nonpolar or weakly polar solvents and showed poor affinity toward such solvents, confining the practical implementation of the composite membrane.^[^ ^58^ ^]^

**Figure 8:**
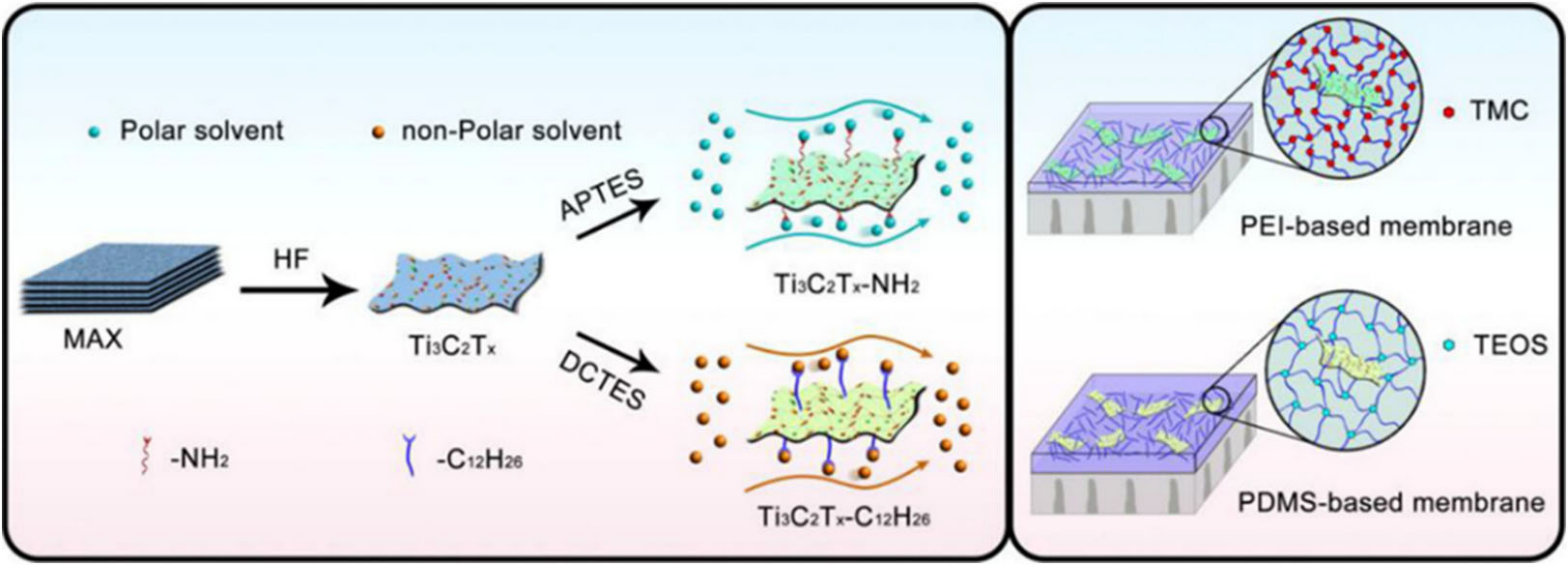
a) Schematic diagram of PEI‐based and PDMS‐based membranes. Reproduced with permission.^[^ ^59^ ^]^ Copyright 2017, Elsevier Ltd.

Grafting different functional groups on MXenes was theoretically feasible for regulating the interaction between targeted solvents and membranes for the sake of specific‐task solvent transport.^[^ ^59^ ^]^ Adopted from previous work, PEI and PDMS acted as polymer matrices with MXene as the nanofiller, whereas hydrophilic –NH_2_, –COOR, and hydrophobic –C_6_H_6_, –C_12_H_26_ were functionalized on MXene (**Figure** **8**a) to separately enhance the flux of polar isopropanol, ethyl acetate, and nonpolar toluene, n‐heptane. A series of tests were conducted to confirm the strongly enhanced affinity, including contact angle, solvent uptake, and area swelling tests, which were highly consistent with the initial hypothesis. Simultaneously, these modified MXene derivates were endowed with an optimum rejection ability due to the extended pathways for solutes. As such, the grafted membrane conquered the trade‐off between selectivity and permeability to some extent, implying a promising future for MXene‐based membranes in solvent purification.

Again, another hydrophilic –NH_2_‐modified Ti_3_C_2_T_*x*_ membrane exerted extremely high permeances of acetonitrile and methanol, as high as 3337 and 3018 L m^−2^ h^−1^ bar^−1^, respectively. These results could be attributed to the high alignment between the polarity of the solvents and hydrophilicity of the membranes; therefore, the aggregates accelerated the transportation in capillary channels. In contrast, when nonpolar solvents are transferred in both hydrophilic and hydrophobic nanochannels, there is no obvious difference because of the out‐of‐order configuration.^[^ ^60^ ^]^

Wang et al.^[18c]^ demonstrated that a regular‐stacked Ti_3_C_2_T_*x*_ membrane with 2 nm interlayer channels imparted high permeance for six solvents, outperforming other laminated membranes in terms of separation ability. According to their report, acetone and acetonitrile had unparalleled permeance of 5000 L m^−2 ^h^−1^ bar^−1^, and the other four solvents obeyed the following order: methanol > ethanol> dimethylformamide > 2‐propanol. The membrane effectively rejected dyes with diameters larger than 2 nm whereas poorly excluded the dyes with diameters of 1.9 nm, indicating the nanochannels were narrowly distributed with a width of 2 nm according to the size exclusion mechanism.^[18c]^

Instead of fabricating membranes with regular nanostructure and flat surface, Xing et al.^[48d]^ designed a kind of crumpled Ti_3_C_2_T_*x*_ laminar membrane with ample interfacial voids through a freeze‐drying method, which demonstrating ultrahigh permeance of water and acetone due to the enlarged channels by the voids. And by varying the thickness of the membrane, the rejection rate for different size molecules could be manipulated, and high preservation could be achieved.

Apart from its outstanding nanofiltration performance, the MXene nanosheets‐based MMMs have been applied for the solvent dehydration process through pervaporation, which permeates solutes via the solution‐diffusion mechanism.^[^ ^61^ ^]^ Cui's^[28c]^ group introduced MXene nanosheets to CS matrix to fabricate MMMs for the dehydration of different solvents. According to water contact angle tests, the hydrophilicity of the membrane kept almost constant after introducing MXene, so the sorption of water remained unchanged. Therefore, the enhanced dehydration performance was attributed to the additional channels brought by MXene laminates, which favored transporting water over solvents. And, the optimal loading of MXene was demonstrated as 3 wt% because the nonselective defects and aggregation started to appear when the loading was increased to 5 wt%, leading to a degraded performance. In addition, the MXene/CS membrane showed excellent stability during 30 h long‐term operation at 50 °C.^[28c]^ The same approach was applied in mixing polyvinyl alcohol (PVA) and MXene to dehydrate ethanol according to a recent article,^[^ ^62^ ^]^ attaining a higher separation factor of 2585 but comparably lower flux. This was ascribed to the elevated cross‐linking density of the composite membrane after embedded with MXene, which resulted the increased impedance of water diffusion. In another different work, the multilayered MXenes, synthesized without long‐time ultrasound, were incorporated with SA^[28d]^ for ethanol dehydration. Because of the greatly improved hydrophilicity, only 0.12 wt% loading of multilayered MXene increased the separation factor from 929.3 to 9946.

In contrast to the aforementioned MMMs, a pristine Ti_3_C_2_T_*x*_ membrane with a thickness of 2 μm prepared via self‐stack process was constructed for alcohol dehydration. When the ethanol weight content was increased from 75% to 95%, the total flux declined by 11.2%, whereas the separation factor was increased by 9 times; even for isopropanol, identical trends could be observed.^[^ ^63^ ^]^

As a less explored MXene member, Ti_2_CT_*x*_, was also used in membrane construction^[^ ^64^ ^]^ for the dehydration of isopropanol. Taking advantage of negatively charged Ti_2_CT_*x*_, hyperbranched poly‐ethylenimine (HPEI) with positive charge was able to intercalate between adjacent interlayers to regulate the stacking behavior. Thereafter, trimesoyl chloride (TMC) was introduced to seal possible nonselective defects by interfacial polymerization with HPEI. Since the hydrophilicity of Ti_2_CT_*x*_ is better than Ti_3_C_2_T_*x*_, the modified Ti_2_CT_*x*_ membrane showed higher selectivity with similar flux under the same condition. In the absence of a sealing layer, their group^[^ ^65^ ^]^ proposed that the polyelectrolytes functionalities were helpful for enhancing separation performance in the isopropanol dehydration process, which was similar to the regulation of physicochemical properties. For 90 wt.% isopropanol/water mixture, a decent separation factor of 1932 and an appealing flux over 1.2 kg m^−2^ h^−1^ were reached at the same time.

### 3.4 Nanofluidic Ion Transport and Osmotic Energy Conversion

**Figure 9:**
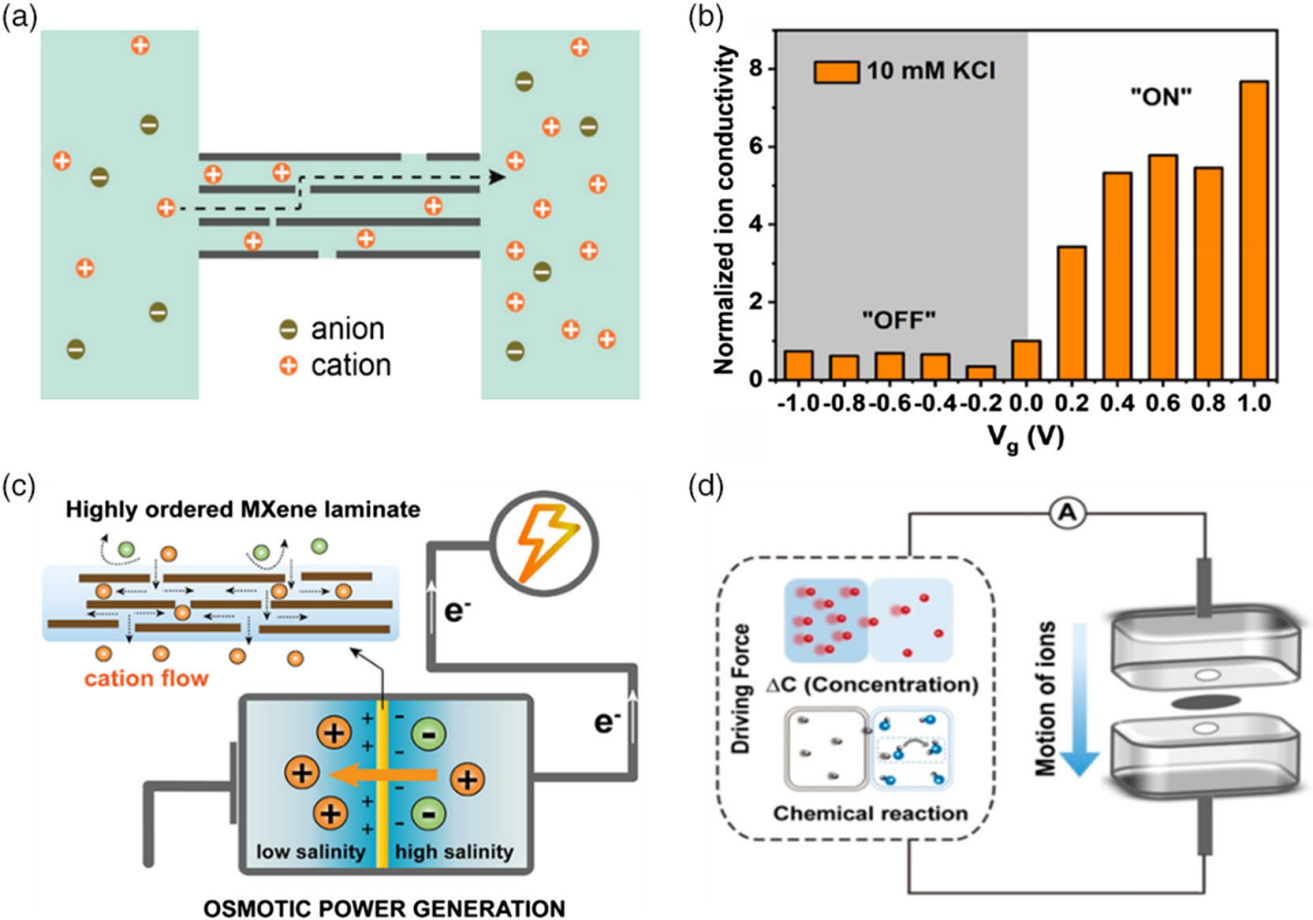
a) Schematic illustration of the nanofluidic device. Reproduced with permission.^[^ ^66^ ^]^ Copyright 2018, American Chemical Society. b) The voltage‐gated ion conductivity of 10 mm KCl. Reproduced with permission.^[^ ^67^ ^]^ Copyright 2019, American Chemical Society. c) Scheme of the Ti_3_C_2_T_*x*_ membrane‐based osmotic energy generator. Reproduced with permission.^[68a]^ Copyright 2019, American Chemical Society. d) The device of osmotic energy capturing assisted with chemical reaction. Reproduced with permission.^[68d]^ Copyright 2020, American Chemical Society.

Utilizing the inherent properties of MXene, such as superior thermal conductivity, electronic conductivity, negative‐charged surface, and nanoscale channels, MXene‐based membranes are attracting growing interest for smart nanofluidic applications. In 2018, Lao and co‐workers^[^ ^66^ ^]^ demonstrated that the Ti_3_C_2_ membrane was a superior nanofluidic platform (**Figure** **9**a) in which ionic transport was governed by surface charge, and the ion conductivity was 1–2 magnitude higher than that in the bulk solution. More importantly, concerning the outstanding photothermal effect, they verified the ionic transport could be completely manipulated by laser light.

Dominated by surface charge, the ionic transport can be manipulated by applying an external voltage to the negative‐charged MXene membranes as well. Ren et al.^[40a]^ first investigated the ions sieving behaviors of laminar MXene membranes. Assuring the ions sieving was under the control of the surface charges, the authors prepared Ti_3_C_2_T_*x*_ nanosheets with a lateral size around 3–15 μm through the mild method for the acquisition of angstrom‐scale channels. As expected, applying negative voltage increased the rejection rate of NaCl and MgSO_4_ because the enhanced interaction between these salts and channels impeded the movement of salts. On the contrary, the application of positive voltage reduced the electrostatic attraction, leading to a decreased rejection. One step further, the MXene membrane was introduced to a voltage‐gated nanofluidic device by Wang and co‐workers for engineering ionic conductivity.^[^ ^67^ ^]^ The voltage‐gating effect was tested to be influenced by the concentrations and species of the solution referring to the literature. As for the 10 mM KCl solution (Figure 9b), they found that the voltage gating on–off ratio was around 10.

Further exploiting the nanoconfined channels and selective ionic transport, MXene‐based membranes were used to harvest osmotic power from salinity gradients.^[^ ^68^ ^]^ Hong et al.^[68a]^ first introduced Ti_3_C_2_T_*x*_ membrane in osmotic energy harvesting (Figure 9c), at a 1000‐fold salinity, they yielded an unprecedently high output energy density of 21 W m^−2^ at room temperature. And, energy density increased to 54 W m^−2^ when the temperature rose to 331 K. Furthermore, MXene/Kevlar nanofiber composite membranes were proven to be high‐performance nanofluidic osmotic power generators, achieving a power density of 4.1 W m^−2^ through blending natural river water and seawater.^[68b]^ Recently, we fabricated negatively and positively charged Ti_3_C_2_T_*x*_ membranes as osmotic energy generators, exhibiting both high power density and energy conversion efficiency due to the abundant surface charges and well‐confined nanochannels.^[68c]^ Taking the forms of concentration gradient into consideration, Liu et al.^[68d]^ used MXene membranes to fabricate a nanofluidic device (Figure 9d) to capture osmotic energy assisted with neutralization reaction and reached a maximum output power density of 7.89 W m^−2^, which outperformed state‐of‐the‐art. The examples aforementioned verified the feasibility and superiority of applying MXene membranes in nanofluidic devices. Moreover, in addition to osmotic energy generators, MXene membranes have great promise in other energy conversion fields, for instance, pressure‐driven electrokinetic power generation.^[^ ^69^ ^]^

## 4 Conclusion and Prospect

In the past decade, 2D materials have emerged as the advanced choices for the fabrication of membranes and enormous progress has been made in the application of the separation process. Compared with other 2D materials, MXenes have many favorable advantages, for instance, the evenly formed surface groups on MXene nanosheets ensure the controllable stacking behavior and uniform channels, and the good hydrophilicity facilitates water transport. Moreover, the superb ion sieving capability and ionic conductivity render MXene a promising future in nanofluidic devices. Though currently the MXene‐based membranes have achieved excellent separation performances, there is still a lot of room for further improvement.

Beginning with the material itself, the majority of existing MXenes were synthesized via top–down method at the current stage, typically, by etching the MAX phases. However, the usage of highly toxic fluorine‐based etching agents and long‐time ultrasound is usually unavoidable, which is not environmentally compatible and is quite time consuming. Conventionally, the etchants are not able to delaminate the sheets completely, so it is unavoidable to use intercalants or sonication, which induces additional damage to nanosheets, such as breakage and pores. To make the production of MXene nanosheets more controllable, for example, adjustable lateral size and number of layers on demand, herein, it is vitally important to realize fluorine‐free etchants and a less complicated process but make sure of the quality and scalability. Moreover, the control of the functionalities’ uniformity in aqueous solution is required for further enhancement as membrane affinity for targeting permeants could be manipulated via modification or cross‐linking protocols.

Second, at present, the preparation of MXene‐based membrane is dominated by the VAF assembly, which is restricted by scalability and precise control of stacking behavior, only suitable for lab‐scale fabrication. Irregularly stacked nanosheets are likely to give rise to unevenly width distribution of nanochannels and undesirable pores, directly influencing the separation performance. Although in the membrane separation field, there is no report about drilling pores on MXene nanosheets for permeating targeted solutes, we desire membranes with such controllable nanostructure, nano‐ or sub‐nano channels. In addition, it is notable that except Ti_3_C_2_T_*x*_, other members of the MXene family remain almost unexplored and over 30 kinds of MXenes have been proposed experimentally or theoretically. Expanding the variety of membranes with unique physiochemistry may promote the elimination of the trade‐off relationship between permeability and selectivity. In a word, the controllability, reproducibility, and scalability of the MXene‐based membrane synthesis need further enhancement.

Third, regardless of various strategies for performance improvement, such as modification, grafting, and cross‐linking (or self‐cross‐linked), we should prioritize stimulating and utilizing the unique chemistry of MXenes. Taking the cost, scalability, and technical maturity into consideration, it is more feasible to construct mixed matrix membranes which incorporate MXene nanosheets as nanofillers to engineering the transport behavior. However, the compatibility of the matrix and filler and the aggregation prevention must be considered. In addition, the stability of the membrane in solvent solution (organic or aqueous), and even in extreme conditions, is equally critical in practical applications, signifying that a noticeable performance fluctuation should not emerge in long‐term operation.

Furthermore, the understanding of the intricate mechanisms of mass transfer in these carefully designed nano‐ or sub‐nanometer channels of MXene‐based membranes for different permeants, such as gas molecules, ions, and solvents, is still in its infancy and necessitates more research efforts. With the aid of computational investigations such as MD simulations and density function theory, these mechanisms should be more clearly elucidated to facilitate the design of advanced membranes with specific channels.

In comparison to solvent purification, the application in gas separation is relatively deficient and less explored, implying that there is sufficient room to extend in this field. Comparing MXene‐based membrane with other well‐established 2D materials, it is essential to understand the inherent property and mechanism of MXenes to achieve optimized utility. Notably, beyond separation applications, the remarkable nanofluidic ion transport and high conductivity of MXenes should be developed, which could be useful in energy conversion systems, energy storage, and other related fields.

In conclusion, due to their attractive properties and fascinating structures, MXenes have received tremendous attention in versatile fields. In this review, we systematically discussed the fabrication of MXene membranes and their applications in separation processes. A concise introduction of MXenes was presented first, the synthesis of MXene nanosheets and fabrication of MXene‐based membranes were then described, and the recent research progresses achieved in gas separation, water treatment, organic solvent purification, emerging nanofluidic ionic transport, and osmosis energy harvesting were discussed in detail. Despite we point out several limitations, we firmly believe that MXene is one of the most promising candidates for membrane technology development.

## Conflict of Interest

The authors declare no conflict of interest.
